# Unilateral Duplication of the Internal Auditory Canal: Stenosis and Incomplete Transverse Megacrest in a Pediatric Patient

**DOI:** 10.7759/cureus.74000

**Published:** 2024-11-19

**Authors:** Brittany Q Dang, Abel Abebe, Alvin Camacho

**Affiliations:** 1 Radiology, University of Texas Medical Branch, Galveston, USA

**Keywords:** duplicated internal auditory canal, pediatric neuroradiology, sensorineural hearing loss, stenotic internal auditory canal, temporal bone anomaly

## Abstract

Based on the available literature, duplicated internal auditory canals (DIACs) represent an exceedingly rare temporal bone anomaly that can result in sensorineural hearing loss (SNHL) in the pediatric population. Often associated with a hypoplastic or aplastic cochlear nerve, DIAC poses limitations on treatment options, such as cochlear implants, for affected patients. Accurate diagnosis and optimal management necessitate a thorough assessment of inner ear structures and potential neural abnormalities with high-resolution computed tomography and magnetic resonance imaging of the temporal bones. However, diagnosis of DIACs poses a significant challenge and may be underrecognized during imaging procedures due to similarities with non-duplicated stenotic cochlear abnormalities and unfamiliar characteristics. This case report describes pertinent radiologic features of DIAC in the case of a pediatric patient who presented with SNHL during late childhood and was subsequently found to have an incomplete bony megacrest within the internal auditory canal, resulting in a unilateral DIAC.

## Introduction

Narrow internal auditory canals (IACs) represent a significant anomaly of the temporal bone, which can lead to sensorineural hearing loss (SNHL) in the pediatric population that can occur sporadically or be associated with specific genetic conditions. Notably, patients with narrow IACs frequently present with dysplastic cochlear nerves, potentially benefitting from therapeutic interventions such as cochlear implants. However, it is observed that pediatric patients displaying SNHL alongside narrow IACs are more likely to exhibit associated inner ear anomalies [1]. This association may impose limitations on available treatment options. Therefore, a comprehensive investigation of the inner ear structures and potential abnormalities through radiologic imaging is crucial for accurate diagnosis and optimal management.

Upon confirmation of SNHL by audiological assessment, it is recommended that imaging studies utilizing computed tomography (CT) and magnetic resonance imaging (MRI) be conducted to determine cochlear implant candidacy [2]. CT facilitates the visualization of bony irregularities of the inner ear, such as stenotic IAC. While CT is appropriate and well suited for evaluating the inner ear in cases of SNHL, it does not provide the necessary information about neural anatomy. It is imperative to supplement CT with MRI to identify any concomitant neural abnormalities in patients with SNHL [2]. MRI aids in the detection of hypoplastic or aplastic vestibulocochlear nerves in patients with narrow IAC. Presently, the combined use of MRI and CT is considered indispensable for accurate preoperative diagnosis and treatment.

Duplicated IACs (DIACs) represent a particularly uncommon form of inner ear anomaly associated with stenotic IAC and SNHL. The prevalence of DIAC in the general population and among patients presenting with SNHL has not been empirically reported in the literature. However, a study in South Korea that evaluated over 64,000 patients with SNHL estimated a DIAC rate of occurrence of 0.19% within their study group [3]. Further epidemiological studies are needed to ascertain the prevalence of DIAC as the current data are not sufficient for comprehensive understanding. In contrast to non-duplicated stenotic IACs presenting with a dysplastic cochlear nerve, DIACs often present with an aplastic cochlear nerve, rendering them unsuitable for treatment with a cochlear implant. Only a limited number of DIAC cases have been documented in the literature [3,4], and any such cases are considered exceedingly rare. The present case reports a pediatric patient with unilateral DIACs and hypoplastic cochlear nerve presenting as severe SNHL in late childhood.

## Case presentation

An 11-year-old male with a past medical history of attention-deficit disorder, eczema, allergies, and asthma presented with complete unilateral SNHL of the right ear for approximately eight months. The onset of right-sided hearing loss was initially identified during a routine well-child-care appointment. Notably, the patient’s mother observed that he habitually spoke at a higher volume relative to others for over two years despite passing a hearing screening one year prior to diagnosis. The patient was born at term without complications and successfully passed the initial newborn hearing screening. Of clinical relevance, the patient had a history of developmental speech articulation disorder at the age of three, necessitating a six-month intervention by a speech pathologist who did not document any concerns regarding his hearing. His previous speech articulation disorder was suggested to have clinical implications on his auditory health growing up. However, the speech pathologist had consistent documentation of normal auditory examinations annually since, reassuring that his auditory health to this point was normal. There was no history of ear infections, associated symptoms, or family history of conditions commonly associated with hearing or vision loss. Otoscopic examination revealed an anatomically normal external auditory canal and intact bilateral tympanic membranes. Audiological evaluation corroborated complete SNHL in the right ear and normal hearing in the left ear.

**Figure 1:**
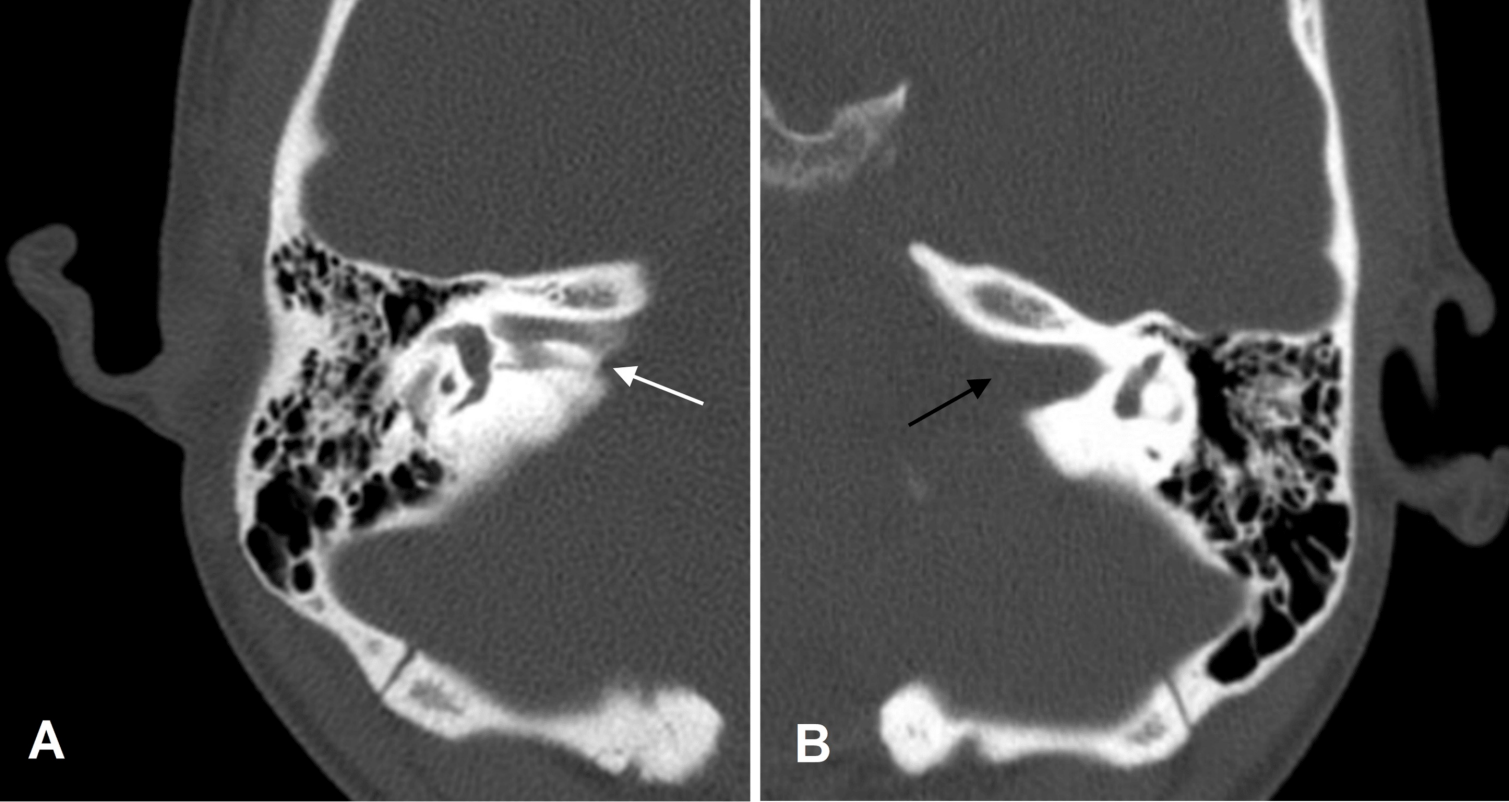
CT temporal bone axial view: the right temporal bone shows (A) a bony prominence (white arrow) within the IAC and (B) a normal, patent IAC on the left side (black arrow) CT: computed tomography; IAC: internal auditory canal

**Figure 2:**
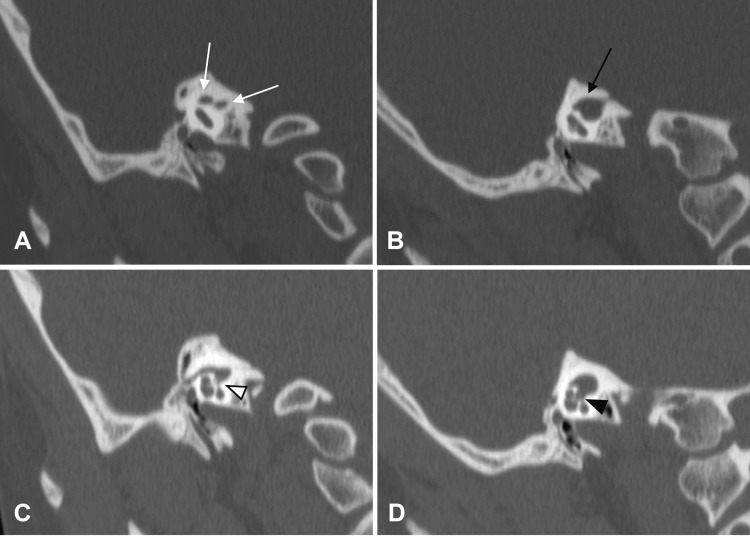
CT of the temporal bones: the right temporal bone in Pöschl view demonstrates (A) a double-barreled IAC (white arrows) and (C) poor communication with the cochlea through the cochlear aperture (white arrowhead). The left temporal bone in Pöschl view shows (B) a single, patent IAC (black arrow) and (D) clear communication with the cochlea via a patent cochlear aperture (black arrowhead) CT: computed tomography; IAC: internal auditory canal

Radiological assessment through CT of the temporal bone using thin 0.6-mm slices was used to visualize the bony anatomy of the temporal bones. Axial (Figure 1) and Pöschl (Figures 2A, 2B) views revealed a stenotic IAC separated into an anterosuperior canal and a narrower posteroinferior canal by an incomplete bony septum on the right side and a single, patent IAC on the left side. Pöschl views on CT also showed a minimally patent cochlear aperture on the right compared to the left (Figures 2C, 2D).

**Figure 3:**
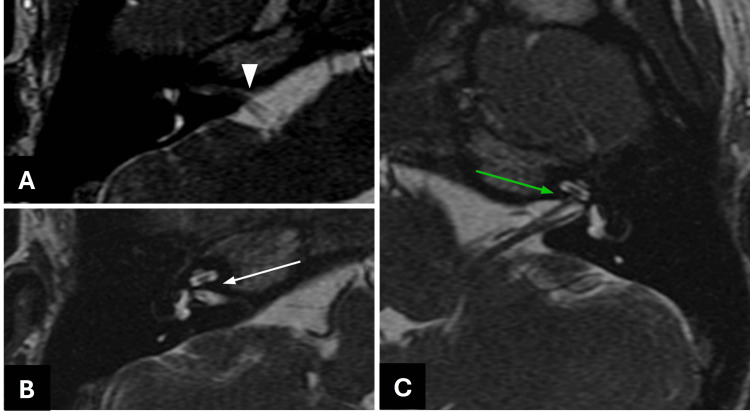
MRI temporal bone axial view on FIESTA-C sequence. (A) Facial nerve and hypoplastic vestibulocochlear nerve entering the narrow anterosuperior segment (white arrowhead) of the right-sided DIAC. (B) Preservation of right IAC fundus (white arrow) distal and inferior to the entry of the nerves is present. (C) Left cochlear nerve traversing the IAC and entering the cochlea through the cochlear aperture (green arrow) MRI: magnetic resonance imaging; IAC: internal auditory canal; DIAC: duplicated internal auditory canal; FIESTA-C: fast imaging employing steady-state acquisition with contrast

**Figure 4:**
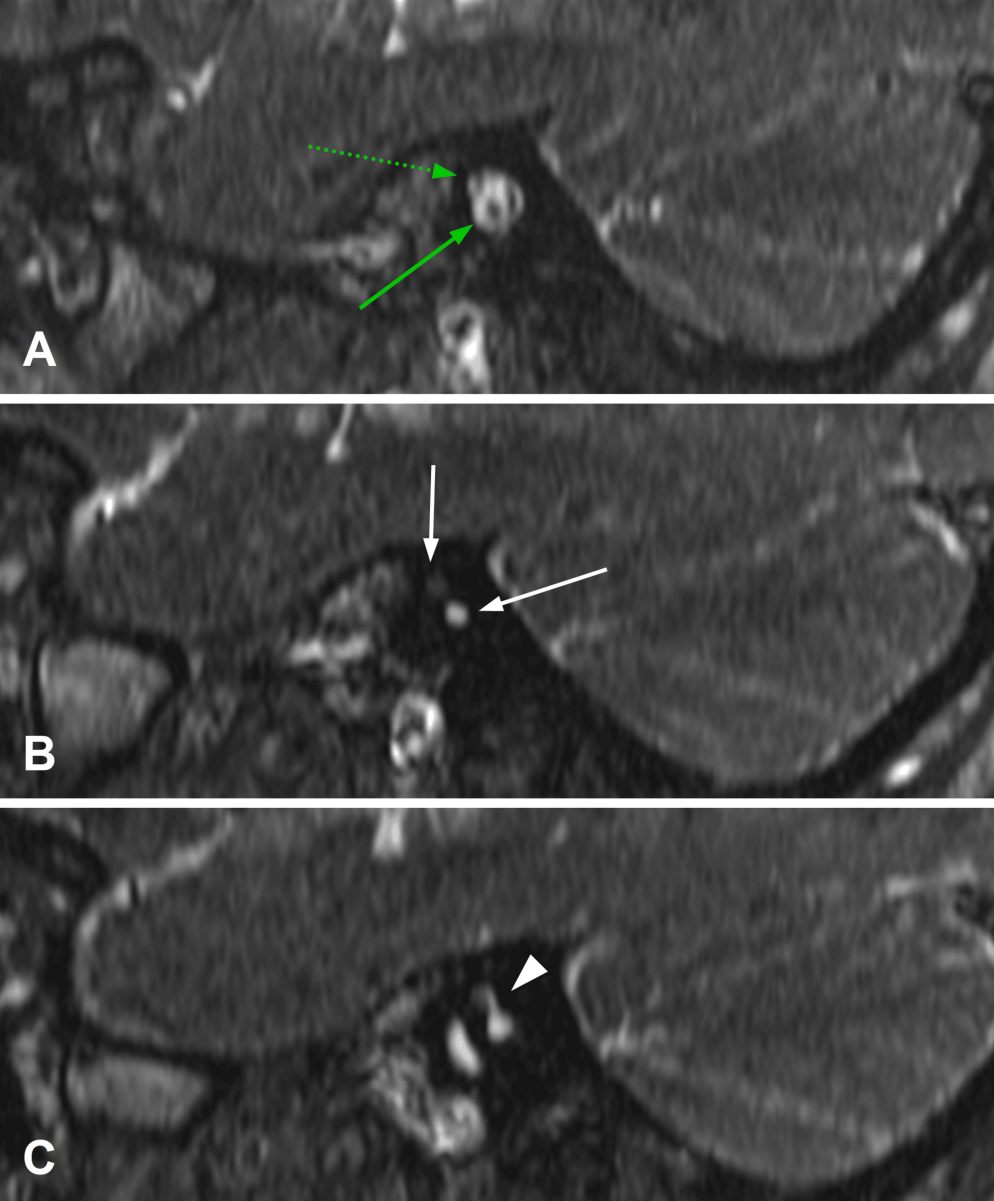
MRI oblique sagittal views: (A) apparent facial (dotted green arrow) and cochlear nerve (solid green arrow) traversing the left IAC; (B) duplication of the IAC on the right side without visualization of the cochlear nerve (white arrows); (C) distal merging of the previously separate DIAC forming the fundus MRI: magnetic resonance imaging; IAC: internal auditory canal; DIAC: duplicated internal auditory canal

A 1.5 Tesla MRI was used to acquire thin 0.5-mm slices of the brain and neural morphology traversing the IAC. On MRI, axial (Figure 3A) views demonstrated the facial and severely hypoplastic vestibulocochlear nerve entering the anterosuperior segment of the DIAC on the right. Preservation of the right IAC fundus distal to the bony megacrest was noted just inferior to the entry point of the nerves (Figure 3B). The left cochlear nerve was observed to enter the left IAC and enter the cochlea through the cochlear aperture (Figure 3C). Oblique sagittal views disclosed normal neural anatomy within the left IAC (Figure 4A). Notably, this view corroborated the presence of a narrow anterosuperior canal and posteroinferior canal (Figure 4B) as well as distal preservation of the IAC fundus on the right (Figure 4C). There was poor visualization of the right cochlear nerve within the IAC due to stenotic canaliculi. The patient continues to receive ongoing care from audiology and otolaryngology and is presently still being evaluated for potential cochlear implantation.

## Discussion

The normal diameter of the IAC ranges from 2 to 6 mm, with stenosis defined as a diameter less than 2 mm [5]. Stenotic IAC can manifest with or without duplication. Proper differentiation between a stenotic IAC with duplication and a stenotic IAC without duplication is pivotal in determining the suitability of cochlear implants for patients. In the present case, the patient exhibited a narrow right IAC with an incomplete vertical crest separating an anterosuperior portion and a posteroinferior portion while preserving the distal fundus on high-resolution CT. MRI demonstrated an intact right facial nerve and a poorly visualized cochlear nerve, indicating a hypoplastic or aplastic right cochlear nerve. These observations correlate with previous studies [3]. Additionally, in cases of DIAC, akin to our patient’s condition, cochlear implants are less likely to address hearing loss due to the concomitant hypoplastic or aplastic cochlear nerve [3].

The prevailing pathogenesis theory of IAC stenosis postulates that the cochlea stimulates the growth of the vestibulocochlear nerve, around which the bony IAC is formed. However, if the vestibulocochlear nerve exhibits hypoplasia or aplasia during development, chondrification and ossification of the AIC may be compromised, resulting in stenotic IAC formation [6]. Importantly, the facial nerve initially evolves independently and is subsequently encompassed by the canal formation around the vestibulocochlear nerve. This developmental process may occasionally lead to IAC duplication in instances where the IAC is narrow with a hypoplastic or aplastic vestibulocochlear nerve [4].

There are only a handful of cases in the literature documenting unilateral DIAC in pediatric patients with SNHL [3,4]. These cases have identified that DIACs are frequently associated with varying degrees of SNHL detected from early to late childhood [3]. Common characteristics of this inner ear anomaly include hypoplastic or aplastic cochlear nerves and preservation of ipsilateral facial nerve function. Additionally, patients may experience associated tinnitus or vestibular symptoms. DIAC is often an isolated occurrence with no family history of SNHL or DIACs. Morphologically, the bony septum can be complete or incomplete, with an anterosuperior portion continuous with the facial nerve canal and a posteroinferior segment communicating with the cochlea and vestibule. Although DIAC may initially be considered a relative contraindication to cochlear implants, there is at least one case documenting a patient with DIAC and hypoplastic cochlear nerves benefitting from cochlear implantation [7].

The diagnosis of DIACs is challenging and may be overlooked during imaging due to similarities with non-duplicated stenotic cochlear abnormalities and unfamiliar features [8]. According to the American College of Radiology Appropriateness Criteria, CT without contrast and MRI with or without contrast are recommended for the evaluation of cochlear implant candidates. High-resolution CT of the temporal bone is utilized to detect bony abnormalities of IACs, while MRI is the standard for assessing the morphology of IAC nerves [1]. These images should be obtained with thin slices (0.5-0.6 mm) to ensure high resolution, especially in pediatric patients. The complexity of the megacrest forming the DIAC necessitates the use of multiple views on both CT and MRI for accurate diagnosis. Key views that may be most helpful for detecting this anomaly include axial and Pöschl views on CT and axial and oblique sagittal views on MRI fast imaging employing steady-state acquisition with contrast (FIESTA-C) sequence. The axial view on CT provides a cross-sectional perspective of the horizontal path of the IACs, enabling a comparison of both IACs and identification of any bony crests or duplicated canals, particularly when segmented into anterior and posterior parts. However, diagnosis of DIAC with axial views alone may be challenging when segmented into superior and inferior portions, which can be superimposed and appear as a single narrowed canal axially. The Pöschl view, perpendicular to the longitudinal axis of the temporal bone, assists in identifying the extent of the bony megacrest, confirming two auditory canals, and visualizing the patency of the cochlear aperture. Axial views on the FIESTA-C sequence are essential for evaluating the condition of the ipsilateral vestibulocochlear and facial nerves. Additional oblique sagittal views in this sequence serve as a window to confirm the presence of the expected facial, cochlear, superior vestibular, and inferior vestibular nerves within the IAC. The identification of two stenotic canals in this view supports the diagnosis of DIACs [4]. In our case, these views were most useful in identifying the incomplete septum that separated the right IAC into two canals. Other specialized views, such as Stenver’s view, may also provide additional information about the extent of the crest and how the two canals are structured.

## Conclusions

This case report contributes to the limited body of literature concerning DIAC in pediatric patients with severe SNHL. It is imperative to consider this rare anomaly in cases of unilateral SNHL. A comprehensive assessment comprising detailed past medical history, physical examination, audiological assessment, high-resolution CT, and high-resolution MRI of the temporal bones is essential. Accurate diagnosis of this anomaly and identification of a hypoplastic or aplastic cochlear nerve are valuable as it significantly impacts treatment planning for cochlear implant candidates. Therefore, discernment of this anomaly from other inner ear abnormalities is recommended, and the utilization of axial and oblique sagittal views on high-resolution submillimeter MRI FIESTA-C sequence in conjunction with axial and Pöschl views on high-resolution CT may be most beneficial in this regard. Further research into the diagnostic and treatment methods for patients with DIAC is needed for improved management and understanding of this rare condition.
